# Novelties in Brazilian *Tradescantia* L. (Commelinaceae)

**DOI:** 10.3897/phytokeys.80.12232

**Published:** 2017-04-25

**Authors:** Marco O. O. Pellegrini, Rafaela C. Forzza, Cassia M. Sakuragui

**Affiliations:** 1 Universidade de São Paulo, Departamento de Botânica, Rua do Matão 277, CEP 05508-900, São Paulo, SP, Brazil; 2 Jardim Botânico do Rio de Janeiro, Rua Pacheco Leão 915, CEP 22460-030, Rio de Janeiro, RJ, Brazil; 3 Universidade Federal do Rio de Janeiro, CCS, Instituto de Biologia, Departamento de Botânica, Av. Carlos Chagas Filho 373, Bloco A, Sala A1-088, Ilha do Fundão, CEP 21941-902, Rio de Janeiro, RJ, Brazil; 4 Current address: Smithsonian Institution, NMNH, Department of Botany, MRC 166, P.O. Box 37012, Washington D.C. 20013-7012, USA

**Keywords:** *Austrotradescantia*, Commelinales, *Mandonia*, Tradescantiinae, spiderworts

## Abstract

We present a new record to the Brazilian territory (i.e. *Tradescantia boliviana*), the rediscovery of a species exclusively known from the cultivated type collection (i.e. *T. valida*), the description of a new taxon (i.e. *T. chrysophylla*), synonyms for *T. crassula* and *T. boliviana*, correct the typification of *T. crassula*, and designation of a lectotype for *T. ambigua* and T. ambigua var. pilosula. Furthermore, we present illustrations, comments, distribution maps, and identification keys for the studied taxa.

## Introduction

*Tradescantia* L., as currently circumscribed, is the second largest genus of Commelinaceae, with ca. 80 species (Faden 1998; eMonocot 2010; The Plant List 2013). The genus is closely related to *Callisia* Loefl., *Elasis* D.R.Hunt, *Gibasis* Raf., and *Tripogandra* Raf. (Evans et al. 2003; Wade et al. 2006; Burns et al. 2011; Hertweck and Pires 2014), with the genera being differentiated based on inflorescence, floral, and seed morphology, and some additional anatomical characters (Faden and Hunt 1991; Faden 1998; Pellegrini 2015). *Tradescantia* is characterized by its main florescences composed of a double-cincinni fused back to back, with each cincinni being contracted and subtended by an expanded bract, actinomorphic flowers with six equal or subequal stamens, and seeds with a linear hilum (Faden 1998; Panigo et al. 2011; Pellegrini 2015). The genus is currently circumscribed into 12 taxonomic sections and four series, and native to the Neotropics, but with a center of diversity in Mexico and Southern USA. This can be explained by the occurrence of two species-rich sections (T. sect. Tradescantia and T. sect. Setcreasea) in drier environments (Hunt 1975, 1980, 1986a).

In Brazil, *Tradescantia* is represented by four of the 12 taxonomic sections (i.e., T. sect. Austrotradescantia, T. sect. Campelia, T. sect. Mandonia, and T. sect. Zebrina) and 12 species (Pellegrini et al. 2015; Pellegrini 2016, 2017). Currently, nine out of 12 species known to Brazil are circumscribed in T. sect. Austrotradescantia (Pellegrini 2015, 2017), but three species belong to different sections; *T. ambigua* Mart. *ex* Schult. & Schult.f. (T. sect. Mandonia), which is restricted to the Caatinga and Cerrado domains, *T. zanonia* (L.) Sw. (T. sect. Campelia), which is geographically disjunct between the Amazon and the Atlantic Forest domains, and *T. zebrina* Heynh. *ex* Bosse (T. sect. Zebrina), an aggressive invasive species widely distributed in Brazil (Pellegrini 2017). The systematics, taxonomy, and species delimitation in this genus are complex, and have been the subject of many recent studies (Hertweck and Pires 2014; Pellegrini 2015, 2016; Pellegrini et al. 2015, 2016), shedding new light on this challenging group. As part of our ongoing studies in *Tradescantia* (Pellegrini 2015, 2016, 2017; Pellegrini et al. 2015, 2016), we describe a new species for Southern and Southeastern Brazil, present the rediscovery of a poorly understood species, document a new record for the Brazilian territory, and contribute with two new synonyms, as well as comments, illustrations, maps, conservation assessments, and the necessary typifications.

## Methods

The descriptions and phenology of the species were based on herbaria, spirit, fresh material, field data, and literature. All species were studied in the field and thus their descriptions are complemented with field notes, photographs, and cultivated specimens, gathered during field trips throughout Brazil between the years of 2008–2016. Specimens collected by the authors were kept in cultivation at the greenhouse of the Jardim Botânico do Rio de Janeiro, in order to better observe, photograph, and analyze fresh flowers, fruits and seeds, as well as other phenological data. Specimens from the following herbaria were also analyzed: ALCB, B, BHCB, BHZB, BM, BOTU, BRIT, C, CAL, CEPEC, CESJ, CGE, CGMS, CNMT, COR, CVRD, EAC, ESA, F, FCAB, FLOR, FURB, GUA, HAMAB, HAS, HB, HBR, HDCF, HRB, HSTM, HUEFS, HURB, IAC, ICN, INPA, JOI, K, L, M, MBM, MBML, MG, MO, MY, NY, P, PMSP, R, RB, RFA, RFFP, SP, SPF, U, UEC, UFRN, UPCB, US, W, WAG, and WU (herbaria acronyms according to Thiers 2017). The distribution of *Tradescantia boliviana* (Hassk.) J.R.Grant was modified from Grant (2004) with the new Brazilian records. The classification of the vegetation patterns follows IBGE (2012). The indumenta and shapes terminology follows Radford et al. (1974); the inflorescence terminology and morphology follows Weberling (1965, 1989) and Panigo et al. (2011); the fruit terminology follows Spjut (1994); seeds terminology follows Faden (1991); and general terminology for *Tradescantia* follows Pellegrini (2015, 2016) and Pellegrini et al. (2016). Conservation assessments were carried out following IUCN Red List Categories and Criteria, Version 3.1 (IUCN 2001). GeoCAT (Bachman et al. 2011) was used for calculating the Extent of Occurrence (EOO) and the Area of Occurrence (AOO).

## Results

We update the number of species of *Tradescantia* known from Brazil to 14 (from 12), with the description of a new species, a new record, two new synonyms, and the rediscovery of *T. valida* G.Brückn in the wild. We present complete descriptions of the new species and the rediscovered taxon, and detailed diagnoses for the other three studied species. The results are organized in the treated sections (i.e. T. sect. Austrodescantia, T. sect. Campelia, T. sect. Mandonia, and T. sect. Zebrina). Furthermore, as a result of our improved knowledge of *Tradescantia*, we present an updated, illustrated, and more functional identification key for the sections occurring in Brazil.

### Updated key to the Brazilian sections of *Tradescantia* (modified from Pellegrini et al. 2015)

**Table:** 

1	Filaments basally densely bearded with long moniliform hairs, connectives rhomboid, anther sacs elliptic, style cylindrical with conical apex, stigma punctate (Figs 1A, 2H, I); embryotega inconspicuous	**Tradescantia sect. Austrotradescantia D.R.Hunt**
–	Filaments medially sparsely bearded with short moniliform hairs, connectives quadrangular to rectangular (Fig. 6D) or sagittate to linearly-tapered (Figs 4C, 7F), anther sacs C-shaped (Fig. 6D) or round (Figs 4C, 7F), style cylindrical throughout, stigma truncate to capitulate (Fig. 6D) or capitate to trilobate (Fig. 4C, D); embryotega conspicuous	**2**
2	Inflorescences sessile, cincinni bracts reduced (Fig. 5D, E); flowers flat, straight at anthesis and pre-anthesis, sepals equal, free (Fig. 6D), stamens equal, filaments coiling at post-anthesis (Fig. 5F), pollen yellow (Fig. 6D), ovary pubescent (Fig. 5F); embryotega dorsal	**Tradescantia sect. Mandonia D.R.Hunt**
–	Inflorescences pedunculate, cincinni bracts spathaceous (Fig. 4C & 7E); flowers tubular, geniculate at anthesis and pre-anthesis, sepals unequal, irregularly fused (Figs 4C, D, 7E), stamens subequal, filaments straight at post-anthesis, pollen white (Fig. 4D), ovary glabrous; embryotega semilateral to lateral	**3**
3	Inflorescences axillary, perforating the leaf-sheath (Fig. 4A); sepals fleshy, vinous and tightly enclosing the capsule (forming a berry-like fruit) (Fig. 4E), petals sessile, rhomboid to obovate, white (Fig. 4C, D), stamens free from the petals	**Tradescantia sect. Campelia (L.C.Rich.) D.R.Hunt**
–	Inflorescences terminal, not perforating the leaf-sheath (Fig. 7E); sepals membranous, hyaline, loosely enclosing the capsule (Fig. 7E), petals clawed, ovate to elliptic, pink to purple (Fig. 7E), stamens epipetalous	**Tradescantia sect. Zebrina (Schnizl.) D.R.Hunt**

#### Tradescantia sect. Austrotradescantia

Taxon classificationPlantaeCommelinalesCommelinaceae

D.R.Hunt, Kew Bull. 35(2): 440. 1980.

##### Diagnosis.

The section is characterized by perennial herbs, with thin and fibrous roots, definite or indefinite base, rhizomes absent, leaves with asymmetric base, inflorescences terminal or at the apex of the stems, pedunculate, cincinni bracts leaf-like or rarely spathaceous, bracteoles inconspicuous, flowers flat, sepals equal, free, generally keeled, petals free, sessile, stamens 6 and equal, free, filaments straight at post-anthesis, basally densely bearded with long moniliform hairs, connectives rhomboid, anther sacs elliptic, ovary glabrous, stigma punctate, seeds costate, rarely rugose, embryotega inconspicuous and dorsal (Pellegrini 2015; Pellegrini et al. 2016).

##### Comments.

Tradescantia sect. Austrotradescantia has been the subject of several recent studies (Pellegrini 2015, 2016, 2017; Pellegrini et al. 2015, 2016; Funez et al. 2016). Species diversity in this section is centered in Southeastern and Southern Brazil, where all of the accepted species occur. However, some species in the group have a wider distribution, reaching neighboring countries like Argentina, Bolivia, Paraguay, and Uruguay (Pellegrini 2015; Pellegrini et al. 2016). Tradescantia sect. Austrotradescantia has recently been revised, and its morphology thoroughly analyzed in a yet unpublished MSc thesis (Pellegrini 2015). As part of our revision of this section, we describe a new species, report the rediscovery and inclusion of *T. valida* in the section, and a new synonym for *T. crassula* (with a correction of its typification).

#### Tradescantia crassula

Taxon classificationPlantaeCommelinalesCommelinaceae

Link & Otto, Icon. Pl. Rar. 2: 13, pl. 7. 1828.

Figs 1 , 8

Tropitria crassula (Link & Otto) Raf., Fl. Tell. 3: 68. 1837. Lectotype (designated here). BRAZIL. Rio Grande do Sul: Rio Pardo, fl., fr., s.dat., F. Sellow 3033 (B barcode B100521014!).
Tradescantia crassipes Graham, Edinburgh New Philos. J. Jan.–March: 388. 1829, nom. nud.
Tradescantia schwirkowskiana Funez et al., Phytotaxa 272 (1): 64. 2016. Holotype. BRAZIL. Santa Catarina: São Bento do Sul, borda da ferrovia às margens do Rio Banhados, fl., fr., 16 Nov 2015, L.A. Funez & P. Schwirkowski 5037 (FURB No. 50791!; isotypes: C n.v., HURB n.v.). **Syn. nov.**

##### Diagnosis.

*Herbs* with a definite base, terrestrial, rupicolous or epiphytes. *Roots* thin, fibrous, cream to light brown, emerging from the basalmost nodes. *Stems* erect, succulent, rarely to densely branched at the base, sometimes branching at the upper half; internodes medium to dark green, glabrous, sometimes with a leaf-opposed longitudinal line of short, uniseriate, light brown to hyaline hairs in the terminal portion of the stems. *Leaves* distichously or spirally-alternate, sessile; sheaths light green, sometimes with green striations, glabrous, margin ciliate to setose, hairs hyaline; blades elliptic to broadly elliptic to ovate to broadly ovate to obovate, rarely lanceolate, falcate to complicate, succulent, glabrous on both sides, adaxially glossy light to medium to dark green, sometimes glaucous, abaxially light to medium green, turning olive-green to greyish green to brown when dry, obtuse to truncate, rarely cuneate, margin green, minutely ciliolate to ciliate, slightly revolute, apex acute to obtuse, rarely acuminate; midvein conspicuous to inconspicuous, adaxially impressed to inconspicuous, secondary veins inconspicuous on both sides, sometimes slightly conspicuous on both sides. *Synflorescences* terminal or axillar in the distal portion of the stems, composed of a solitary main florescence, 1 per leaf axis. *Inflorescences* (*main florescences*) consisting of a pedunculate double-cincinni fused back to back; peduncles green, glabrous, sometimes with a leaf-opposed longitudinal line of short, uniseriate, light brown to hyaline hairs; peduncle bracts absent; supernumerary bracts absent; cincinni bracts similar to each other, rarely unequal or reduced in some axillary inflorescences, broadly ovate to ovate, leaf-like, glabrous, adaxially light to medium to dark green, abaxially light to medium green, base cordate to obtuse, not saccate, margin entire to minutely ciliolate to sparsely setose near the base, apex acute; double-cincinni 8–28-flowered. *Flowers* bisexual, actinomorphic, flat (not forming a floral tube); floral buds broadly ovoid; pedicels green to vinaceous, glabrous, rarely sparsely glandular-pubescent; sepals 3, equal, free, ovate, cucullate, margin hyaline, apex acute, persistent in fruit, dorsally keeled, green, rarely vinaceous, setose, with long hyaline hairs along the keel; petals 3, equal, free, elliptic to ovate, rarely broadly ovate, not clawed (sessile), flat, white; stamens 6, arranged in two series, equal, filaments free from the petals and from each other, filaments straight at anthesis and post-anthesis, basally densely bearded with moniliform hairs, hairs as long as the stamens, white, anthers basifixed, rimose, connective expanded, rhomboid, yellow, anther sacs ellipsoid, divergent, yellow, pollen yellow; ovary sessile, subglobose to globose, white, smooth, glabrous, 3-loculate, locules equal, locule 2-ovulate, ovules uniseriate, style straight, white, cylindrical, conical at the apex, stigma punctate, pistil longer than the stamens. *Capsules* subglobose, light to medium brown when mature, smooth, glabrous, loculicidal, 3-valved, sometimes apiculate due to persistent style base. *Seeds* exarillate, 1–2 per locule, ellipsoid to narrowly trigonal, cleft towards the embryotega, ventrally flattened, testa grey to greyish brown, farinose, costate arranged in ridges radiating from the embryotega; embryotega dorsal, relatively inconspicuous, generally covered by a cream farina, without a prominent apicule; hilum linear, longer than ½ the length of the seed.

**Figure 1.**
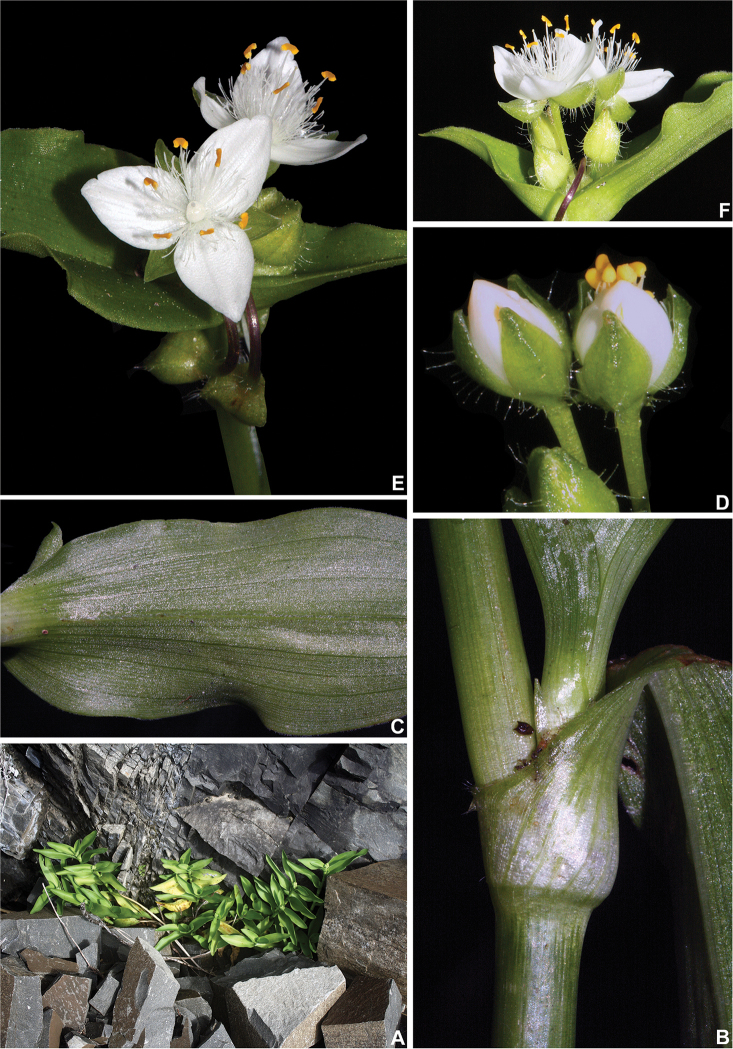
*Tradescantia crassula* Link & Otto. **A** habit, showing the erect stems, and distichously-alternate leaves with conduplicate blades **B** detail of the stem and leaf-sheath **C** detail of the abaxial side of the leaf-blade, showing the lack of hairs and the slightly conspicuous secondary veins **D** detail of floral buds, showing the setose hairs, restricted to the keels of the sepals **E** flower **F** detail of the inflorescence, showing the non-saccate cincinni bracts. Photographs by M.O.O. Pellegrini.

##### Specimens seen.

**ARGENTINA. Misiones**: Cainguás, pequeño campo a la entrada del Salto Golondrina, sobre Arroyo Guiray, fl., fr., 8 Nov 2000, M.E. Múlgura de Romero et al. 2470 (CTES, SI); General Manuel Belgrano, ruta nacional 101, 8 km de Bernardo de Irigoyen hacia San Antonio, Salto Andrecito, fl., 15 Oct 1996, O. Morrone et al. 1393 (CTES, SI). **BRAZIL. Minas Gerais**: Extrema, trilha para a Pedra das Flores, fl., 24 Oct 2009, G.H. Shimizu 226 (RB, UEC). **Rio Grande do Sul**: Barão, sudoeste de Garibaldi, Estrada para Carlos Barbosa, fl., fr., 22 Nov 2005, M.C. Machado & L.Y.S Aona 607 (UEC, HUEFS). **Santa Catarina**: Campo Belo do Sul, Fazenda Gateados, fr., 15 Jul 2008, M. Verdi et al. 2028 (FURB, RB); São Bento do Sul, Floresta Ombrófila Mista, fl., fr., 31 Dec 2013, P. Schwirkowski 197 (FURB). **São Paulo**: Jundiaí, Serra do Japi, fl., fr., 25 May 1994, J. Semir et al. 31648 (UEC); loc. cit., Trilha do Mirante, fl., fr., 18 Jul 1995, R. Mello-Silva et al. 1074 (SPF); loc. cit., Serra do Jundiaí, sentido bairro Eloy Chaves, próximo à represa do DAE, fl., 23 Jan 1998, E.R. Pansarin 136 (SP, UEC); Itararé, Fazenda Ibiti (Ripasa), beira da estrada Itararé-Bonsucesso, fl., fr., 30 Oct 1993, V.C. Souza et al. 4531 (ESA, RB); São Paulo, Cidade Jardim, fl., fr., 11 Mar 1932, W. Hoehne s.n. (IPA 69219, SPF 17149).

##### Distribution and habitat.

*Tradescantia crassula* occurs in Argentina and Brazil (in the states of Minas Gerais, São Paulo, Paraná, Santa Catarina, and Rio Grande do Sul) (Fig. 8). It is commonly found growing in rocky outcrops, grasslands and open areas, under full sunlight, as rupicolous or terrestrial. It is also found on roadsides and within the understory of open forests, as terrestrial or, more rarely, as an epiphyte.

##### Conservation status.

*Tradescantia crassula* possesses a wide EOO (ca. 408,686.868 km^2^). Thus, following the IUCN recommendations (IUCN 2001), it should be considered Least Concern (LC).

##### Nomenclatural notes.

Funez et al. (2016) indicate that Pellegrini (2015) erroneously designated the specimen *Sellow 3033* (B100521014) as the lectotype for *T. crassula*. However, their affirmation is incorrect according to the *Code* since the thesis lacks either a ISSN or an ISBN, and was never distributed to the general public (McNeill et al. 2012, Art. 29.1). The work cited by Funez et al. (2016) was a draft version of the first author’s unpublished M.Sc. thesis, with many incomplete and partially incorrect data (Pellegrini, unpublished data), and therefore is not considered a effective publication by the *Code*. Furthermore, according to Art. 30.8 (McNeill et al. 2012), any thesis published on or after 1 January 1953 and stated to be submitted to a university for the purpose of obtaining a degree, does not constitute effective publication; unless it contains any kind of statement or other internal evidence that it is regarded as an effective publication by its author or publisher. Since no statement is made in the final version of the thesis (i.e. Pellegrini 2015), the publication does not meet any of the *Code*’s requirements, and therefore cannot be treated as effectively published. In the final version, Pellegrini (2015) gives detailed information on the typification of *T. crassula*, which is effectively published here and corrects the typifications by Funez et al. (2016).

The date written on the original label, and treated by Funez et al. (2016) as the collections date, is “Dec. 1836”. Also, it is possible to see in the label the names of Sellow and Humboldt. According to Stafleu and Cowan (1985), Friedrich Sellow lived from 1789–1831, and collected plant specimens in Southern Brazil and Uruguay 1819–1831, funded by Humboldt. This easily explains why both botanists are mentioned in the original label, and why the collector is to be considered as Sellow, instead of Humboldt or both botanists. Furthermore, given that Sellow died in 1831 and his expeditions were done just before his death, it would be impossible for “Dec. 1836” to represent the actual collection date. We believe this date might correspond to the date when this specimen was acquired by Kunth, and placed into his personal herbarium. Finally, Link and Otto (1828) make direct reference to their new species being based on Sellow collections. According to the *Code* (McNeill et al. 2012, Art. 9.2), the *Sellow 3033* (B100521014) specimen is a suitable choice for a lectotype, superseding the lectotypification of the original illustration, done by Funez et al. (2016). The epitypification by Funez et al. (2016) should be disregarded entirely because the original illustration is informative enough to correctly apply the name *T. crassula*. All the diagnostic features of this species (see comments below) are visible and sufficient for appropriate diagnosis, in the original illustration.

##### Taxonomic notes.

The species in this section are especially variable morphologically and when in cultivation or growing in shaded areas they can change their vegetative morphology quite drastically. Few characters in the vegetative organs were observed to be constant in the *T. crassula* group and thus are of little taxonomic relevance. This can be exemplified by the phyllotaxy and pubescence of the leaf-blades, which have been shown to vary greatly due to ecological features (Pellegrini 2015, 2016). On the other hand, the pubescence of the internodes, leaf-sheaths, and of the margin of the leaf-blades were observed to be constant and reliable for species delimitation. As previously indicated in other *Tradescantia* sections (Anderson and Woodson 1935), the pubescence of the pedicels and sepals seem to be highly stable within each species, easily observable, and thus, reliable for species delimitation. As aforementioned, *T. crassula* is highly variable in vegetative morphology. All studied individuals always presented glabrous leaf-blades, setose sepals with long hairs along the keel, and white petals. The specimens cited by Funez et al. (2016) as representing *T. schwirkowskiana*, fit perfectly the circumscription adopted by us for *T. crassula*, showing variation only in the degree of branching of the stems, color of the leaf-blades, and degree of impression of the secondary veins. All of this morphological variation can be easily explained by the ecological features of the area where the specimens were collected (i.e. shaded moist forests in mountainous regions from the state of Santa Catarina). Aside from that, the authors state that *T. crassula* possesses spirally-alternate leaves and a rhizomatous base. While developing our studies for the taxonomic revision of T. sect. Austrotradescantia and a morphological phylogeny for the genus (Pellegrini 2015), we analyzed 50% of the species in the genus and observed that all species of *Tradescantia* produce spirally-alternate leaves when young. This feature is normally lost during development of most species of T. sect. Austrotradescantia, but is always retained by *T. valida* G.Brückn. (see comments below), sometimes retained by *T. cerinthoides* Kunth (Pellegrini 2015, 2016), and rarely retained by *T. crassula* (Pellegrini, pers. obs.). No species in *Tradescantia* were observed to produce rhizomes (Pellegrini 2015). The only known drought resistance strategy observed in the genus was the production of tuberous roots; present in all species of T. sect. Mandonia, T. sect. Parasetcreasea, T. sect. Separotheca, T. sect. Setcreasea and sect. Tradescantia, and exclusively in *T. commelinoides* Schult. & Schult.f. from T. sect. Cymbispatha (Pellegrini 2015). Thus, *T. schwirkowskiana* is here synonymized under *T. crassula*.

#### Tradescantia chrysophylla

Taxon classificationPlantaeCommelinalesCommelinaceae

M.Pell. sp. nov.

urn:lsid:ipni.org:names:60474204-2

Figs 2 , 8

##### Diagnosis.

Similar to *T. cymbispatha* due to its habit with an indefinite base, creeping stems with ascending apex, sessile succulent leaves with flat blades homogeneously covered by indumenta, inconspicuous secondary veins, saccate cincinni bracts, broadly ovoid floral buds, sepals without keels, and pistil the same length as the stamens. It can be differentiated by its velutine to hispid, golden to light brown indumentum covering almost the entire plant, strongly unequal cincinni bracts, and pedicels and sepals glandular-pubescent, or with a mixture of glandular and eglandular hairs.

**Figure 2.**
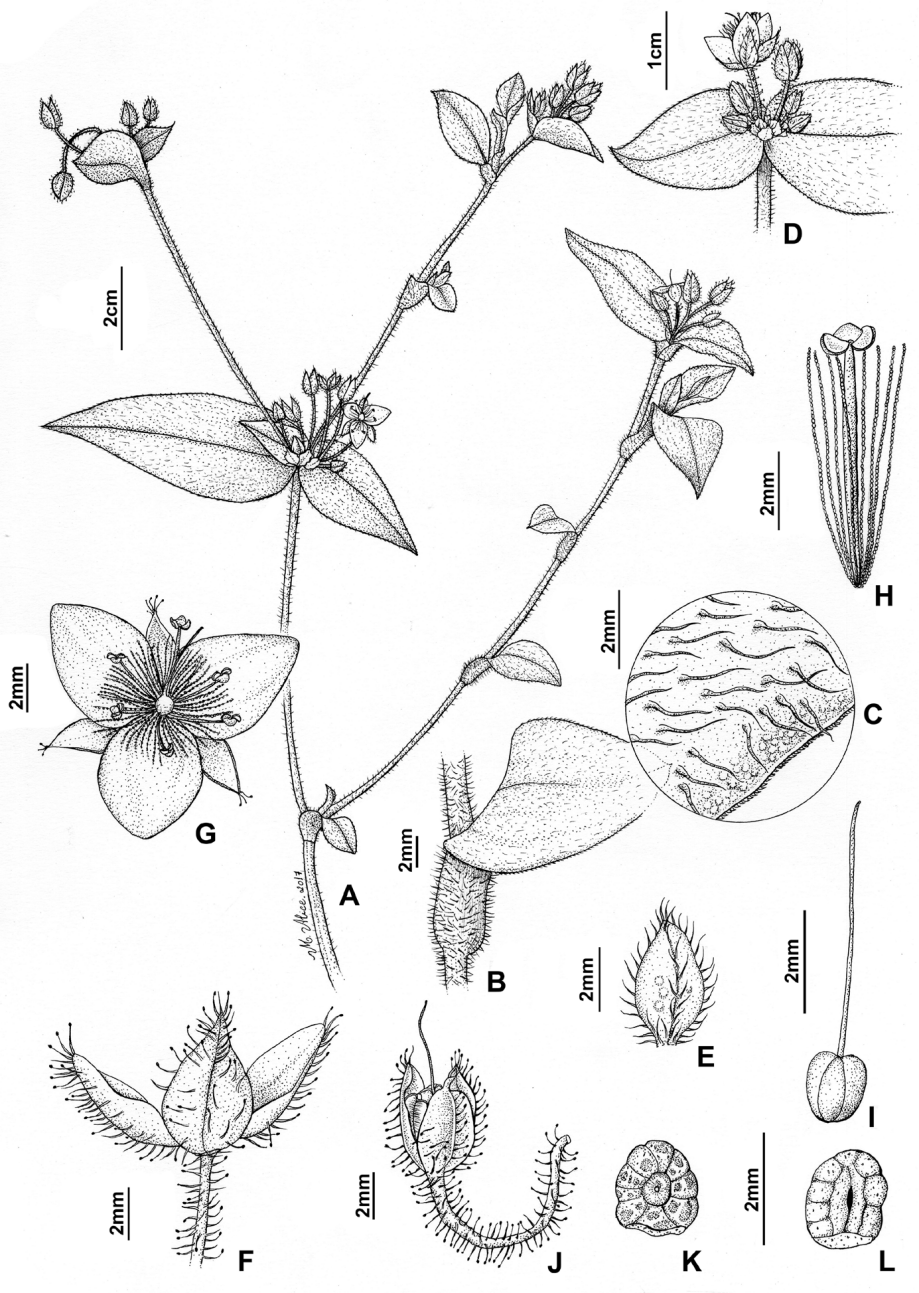
*Tradescantia chrysophylla* M.Pell. **A** habit **B** detail of the stem and leaf-sheath, showing the hispid indumentum, and detail of the sessile leaf-blade **C** detail of the hispid hairs of the leaf-blade and of the ciliolate margin **D** detail of the inflorescence, showing the unequal cincinni bracts **E** detail of the bracteole **F** detail of the pedicel and sepals, showing the glandular hairs **G** frontal view of the flower **H** stamen, showing the filament with basal, dense and long moniliform hairs, the rhomboid connective, and the ellipsoid anther sacs **I** detail of the gynoecium, showing the punctate stigma **J** mature, partially open capsule, still covered by the persistent sepals. **K–L** seed: **K** dorsal view of a seed, showing the costate testa and dorsal embryotega **L** ventral view of the seed, showing the linear hilum. Line drawing by M.A. Rezende.

##### Type.

BRAZIL. São Paulo: Biritiba Mirim, Estação Biológica de Boracéia, fl., 24 Nov 1983, A. Custódio Filho 1910 (holotype: RB!; isotype: SP!).

##### Description.

*Herbs* ca. 11–27 cm tall, with an indefinite base, terrestrial or rupicolous, rarely epiphyte. *Roots* thin, fibrous, cream to light brown, emerging from the nodes touching the substrate. *Stems* creeping to ascending at the apex, delicate to slightly succulent, densely branched; internodes 1.5–8.2 cm long at base, distally shorter, dark green to vinaceous, velutine to hispid, hairs golden to light brown. *Leaves* distichously-alternate, sessile; sheaths 0.4–1 cm long, green to vinaceous with dark green to purple striations, velutine to hispid, margin densely setose, hairs golden; blades broadly elliptic to broadly ovate, 1.8–7.6 × 0.9–3.4 cm, flat, succulent, velutine to hispid on both sides, hairs golden to light brown, adaxially dark green, abaxially vinaceous, turning dark brown to olive-green on both sides when dry, base cordate to rounded, margin ciliolate, apex acute, sometimes acuminate; midvein conspicuous, adaxially impressed, secondary veins inconspicuous, adaxially inconspicuous, abaxially inconspicuous, becoming more evident abaxially when dry. *Synflorescences* terminal or axillar in the distal portion of the stems, composed of a solitary main florescence, 1 per leaf axis. *Inflorescences* (*main florescences*) consisting of a pedunculate double-cincinni fused back to back; peduncles (0.4–)1.1–9.5 cm long, vinaceous, velutine to hispid, hairs golden to light brown; basal bract inconspicuous, tubular, hyaline, glabrous; peduncle bracts absent; supernumerary bracts absent; cincinni bracts strongly unequal among themselves, elliptic to ovate to broadly ovate, 0.9–6.6 × 0.4–3.1 cm, leaf-like, velutine to hispid, hairs golden to light brown, adaxially dark green, abaxially vinaceous, base cordate to round, saccate, margin ciliolate, apex acute; double-cincinni (4–)6–12-flowered; bracteoles inconspicuous, imbricate, linear-triangular to triangular, hyaline. *Flowers* bisexual, actinomorphic, flat (not forming a floral tube), 1.1–1.6 cm diam.; floral buds broadly ovoid, apex acute; pedicels upright at anthesis and pre-anthesis, reflexed at post-anthesis, 0.9–1.3 cm long, glandular-pubescent, rarely with a mixture of glandular and eglandular, golden to light brown hairs; sepals 3, equal, free, ovate, cucullate, margin hyaline, apex acute, persistent in fruit, 4.7–5.8 × 2.6–4 mm, without dorsal keels, glandular-pubescent or with a mixture of glandular and eglandular, golden to light brown hairs; petals 3, equal, free, elliptic to ovate, rarely broadly ovate, not clawed (sessile), flat, 8.8–9 × 5.7–6.2 mm, white; stamens 6, arranged in two series, equal, filaments free from the petals and from each other, filaments 6–6.2 mm long, straight at anthesis and post-anthesis, basally densely bearded with moniliform hairs, hairs as long as the stamens, white, anthers basifixed, rimose, 0.6–0.8 × 0.3–0.7 mm, connective expanded, rhomboid, yellow, anther sacs ellipsoid, divergent, yellow, pollen yellow; ovary sessile, subglobose, 1.5–1.7 × 1.2–1.4 mm, white, smooth, glabrous, 3-loculate, locules equal, locule 2-ovulate, ovule uniseriate, style straight, white, cylindrical, conical at the apex, 4–4.2 cm long, stigma punctate, pistil the same length as the stamens. *Capsules* subglobose to globose, 2.7–3.2 × 2.2–2.8 mm, light to medium brown when mature, smooth, glabrous, loculicidal, 3-valved, sometimes apiculate due to persistent style base. *Seeds* exarillate, 1–2 per locule, 1.1–1.5 × 1.0–1.4 mm, ellipsoid to narrowly trigonal, not cleft towards the embryotega, ventrally flattened, testa grey to greyish brown, farinose, costate arranged in ridges radiating from the embryotega; embryotega dorsal, relatively inconspicuous, generally covered by a cream farina, without a prominent apicule; hilum linear, ½ the length of the seed.

##### Specimens seen

**(paratypes). BRAZIL. Paraná**: Campo Largo, Caverna do Pinheirinho, fl., fr., 13 Oct 1996, G. Tiepolo & A.C. Svolenski 716 (EFC, MBM). **Rio de Janeiro**: Itatiaia, Serra do Itatiaia, Maromba, fl., 23 Oct 1931, C. Porto 2101 (RB). **Santa Catarina**: Rio do Sul, estrada Rio do Sul-Lontras, fl., 3 Dec 2013, A.L. Gasper et al. 3270 (FURB). Rodeio, borda da floresta, fl., fr., 10 Oct 2015, L.A. Funez 4549 (FURB). Urubici, Salto do rio Avencal, fl., fr., 16 Oct 2004, G. Hatschbach et al. 78097 (MBM). São Miguel D’Oeste, forest above rio Reperi-guaçu, Peperi, fl., fr., 21 Oct 1964, L.B. Smith & R. Reitz 12775 (FLOR, HBR, NY, US). **São Paulo**: Jundiaí, mata de planalto na Serra do Japi, fl., 11 Aug 1976, H.F. Leitão-Filho & G.J. Shepherd 2536 (MBM, NY, UEC); loc. cit., ca. 10 km SW de Jundiaí, fl., fr., 8 Oct 1976, H.F. Leitão-Filho et al. 3175 (E, MBM, NY, UEC, UFG, US). Salesópolis, Estação Biológica de Boracéia, estrada para a barragem da SABESP no Rio Guaratuba, fl., 5 Sep 1994, R. Simão-Bianchini et al. 505 (RB, SP, UEC).

##### Etymology.

The epithet “*chrysophylla*” means golden leaves and is given after the golden hairs that cover the whole plant, but especially the leaves.

##### Distribution and habitat.

*Tradescantia chrysophylla* is endemic to Brazil, more precisely to the states of Rio de Janeiro, São Paulo, Paraná and Santa Catarina (Fig. 8). It can be found growing as a terrestrial, rupicolous or as an epiphyte, understory in shaded and moist forests.

##### Phenology.

It was found in bloom and fruit from August to December, but peaking during October.

##### Conservation status.

*Tradescantia chrysophylla* possesses a wide EOO (ca. 173,649.709 km2^2^), but a considerably narrow AOO (ca. 36.000 km^2^). Since it is known from very few and fragmented collections, following the IUCN (2001) recommendations, *T. chrysophylla* should be considered Endangered [EN, A2cde+ B2ab(ii, iii, iv)+D2].

##### Discussion.

*Tradescantia chrysophylla* is morphologically similar to *T. cymbispatha* C.B.Clarke, *T. fluminensis* Vell. and *T. mundula* Kunth due to their indefinite base, creeping stems with ascending apex, saccate cincinni bracts, petals always white, pistil as long as the stamens, seeds with uncleft testa towards the embryotega, and hilum ½ the length of the seed. However, it can be easily differentiated from *T. fluminensis* and *T. mundula* by its sessile succulent leaves, blades homogeneously covered by indumentum, and inconspicuous secondary veins (*vs.* leaves membranous, blades glabrous or unevenly covered by indumentum, and impressed secondary veins), floral buds broadly ovoid (*vs.* ovoid to narrowly ovoid), and sepals without keels (*vs.* keeled sepals). *Tradescantia chrysophylla* is considerably more similar to *T. cymbispatha* due to their sessile, succulent leaves homogeneously covered by indumenta, inconspicuous secondary veins, and sepals without keels. Nonetheless, in *T. chrysophylla* the indumentum is velutine to hispid and golden to light brown (*vs.* strigose and hyaline in *T. cymbispatha*), the cincinni bracts are strongly unequal (*vs.* equal), and the pedicels and sepals are glandular-pubescent with golden to light brown hairs or covered by with a mixture of glandular and eglandular hairs (*vs.* velutine, covered by eglandular hyaline hairs). Furthermore, *T. chrysophylla* can be differentiated from almost all the species of T. sect. Austrotradescantia by its golden to light brown indumentum covering almost the entire plant. The only other species known to possess a similarly colored indumentum is *T. cerinthoides* (Pellegrini 2015, 2016). *Tradescantia chrysophylla* can be easily differentiated by its indefinite habit base (*vs.* definite in *T. cerinthoides*), prostrate stems (*vs.* ascending to erect), saccate cincinni bracts (*vs.* non-saccate), pistil the same length as the stamens (*vs.* longer than the stamens), petals always white (*vs.* ranging from white to pink to lilac), seed not cleft towards the embryotega (*vs.* cleft), and hilum ½ the length of the seed (*vs.* longer than ½ the length).

#### Tradescantia valida

Taxon classificationPlantaeCommelinalesCommelinaceae

G.Brückn., Notizbl. Bot. Gart. Berlin–Dahlem 11: 510. 1932.

Figs 3 , 8

##### Type.

BRAZIL. cult. in Hort. Bot. Münster/W., fl., fr., 28 Apr 1932, s.leg. s.n. (holotype: B barcode B 100296487!).

##### Description.

*Herbs* ca. 30–70 cm tall, with indefinite base, rupicolous, rarely terrestrial. *Roots* thin, fibrous, cream to light brown, emerging from the nodes touching the substrate. *Stems* erect, succulent, little branched only at the base; internodes 1.8–7 cm long at base, distally shorter, green, sometimes with vertical reddish purple striations, glabrous. *Leaves* spirally-alternate, sessile; sheaths 0.4–1.8 cm long, light green, sometimes with vertical green or reddish purple striations, glabrous, margin setose, with long hyaline hairs; blades linear elliptic to linear lanceolate to lanceolate, rarely ovate, 2.7–18.2 × 1.1–2.5 cm, falcate to complicate, succulent, glabrous, adaxially light to medium green, abaxially light green, rarely tinted vinaceous to completely vinaceous, turning olive-green to light brown when dry, base truncate to obtuse, margin green to vinaceous, setose at base or until the middle with long hyaline hairs, slightly revolute, apex acute to acuminate; midvein conspicuous to inconspicuous, secondary veins inconspicuous, becoming more evident on both sides when dry. *Synflorescences* terminal or axillar in the distal portion of the stems, composed of a solitary main florescence, 1 per leaf axis. *Inflorescences* (*main florescences*) consisting of a pedunculate double-cincinni fused back to back; peduncles 3.5–52 cm long, green, glabrous; basal bract inconspicuous, tubular, hyaline, glabrous; peduncle bracts absent; supernumerary bracts present, 1–3 per inflorescence, similar in shape and size to the cincinni bracts; cincinni bracts unequal among themselves, lanceolate to ovate, rarely broadly ovate, 1–3.5 × 0.3–1.2 cm, spathaceous, glabrous, light-green, abaxially slightly lighter, base truncate to obtuse, not saccate, margin green, setose at base or until the middle with long hyaline hairs, apex acute; double-cincinni (4–)6–26-flowered; bracteoles inconspicuous, imbricate, linear-triangular to triangular, hyaline. *Flowers* bisexual, actinomorphic, flat (not forming a floral tube), 1–1.5 cm diam.; floral buds broadly ellipsoid, apex acuminate; pedicels upright at anthesis and pre-anthesis, reflexed at post-anthesis, 0.7–2 cm long, green, glabrous, rarely sparsely glandular-pubescent, if present hairs hyaline; sepals 3, equal, free, ovate, cucullate, margin hyaline, apex acute, persistent in fruit, 4.8–7.3 × 1.5–3 mm, green, without dorsal keels, glabrous, rarely sparsely pilose at the apex, hairs eglandular, hyaline; petals 3, equal, free, elliptic to ovate, rarely broadly ovate, not clawed (sessile), flat, 5.2–8.6 × 2.7–5.4 mm, white to white with pink apex to light pink; stamens 6, arranged in two series, equal, filaments free from the petals and from each other, filaments 2.8–5 mm long, straight at anthesis and post-anthesis, basally densely bearded with moniliform hairs, hairs as long as the stamens, white, anthers basifixed, rimose, 0.8–1 × 1–1.2 mm, connective expanded, rhomboid, yellow, anther sacs ellipsoid, divergent, yellow, pollen yellow; ovary sessile, subglobose to globose, 1–1.7 × 1–1.3 cm, white, smooth, glabrous, 3-loculate, locules equal, locule 2-ovulate, ovule uniseriate, style straight, white, cylindrical, conical at the apex, 4–5.8 cm long, stigma punctate, pistil longer than the stamens. *Capsules* subglobose to broadly oblongoid, 2.8–4.2 × 1.8–3 mm, light to medium brown when mature, smooth, glabrous, loculicidal, 3-valved, sometimes apiculate due to persistent style base. *Seeds* exarillate, 1–2 per locule, 1.4–3 × 1–1.8 mm, ellipsoid to trigonal, cleft towards the embryotega, ventrally flattened, testa grey to greyish brown, farinose, costate arranged in ridges radiating from the embryotega; embryotega dorsal, relatively inconspicuous, generally covered by a cream farina, without a prominent apicule; hilum linear, longer than ½ the length of the seed.

**Figure 3.**
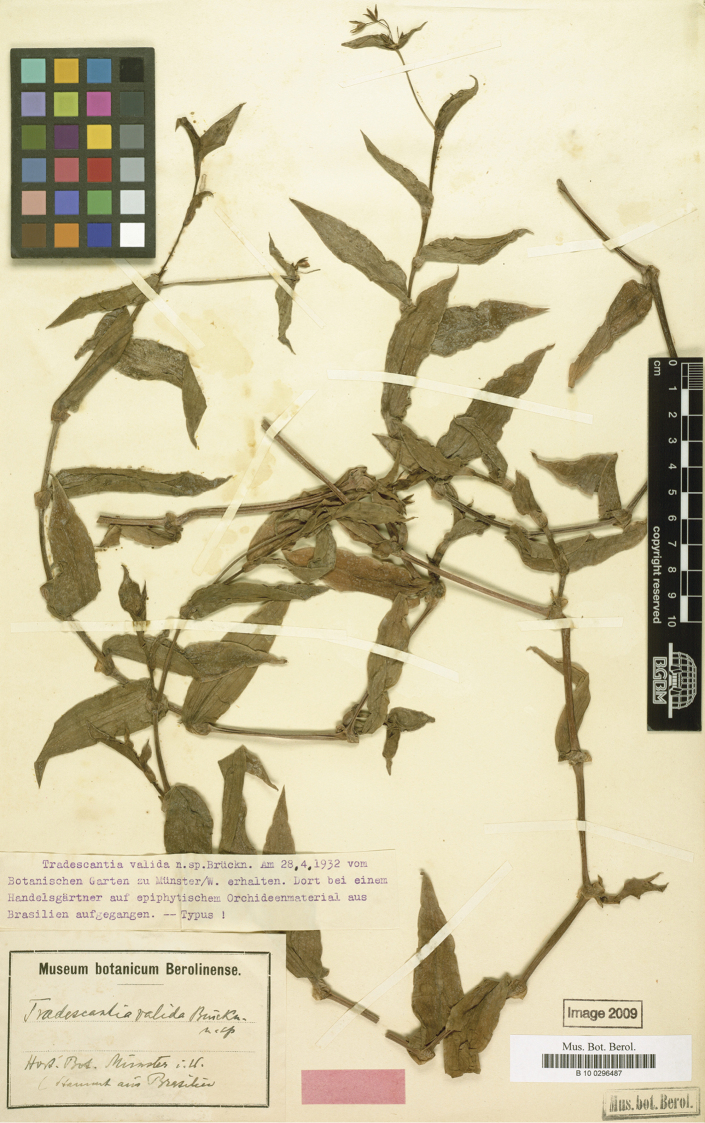
*Tradescantia valida* G.Brückn. Holotype of *T. valida* (B barcode B100296487). Photograph courtesy of the Botanic Garden and Botanical Museum Berlin-Dahlem, Freie Universität Berlin.

##### Specimens seen.

**BRAZIL. Rio Grande do Sul**: Jaguari, ca. 12.5 km ao norte de Jaguari na BR-287 em direção a Santiago, fl., fr., Dec 2005, L.Y.S. Aona & M.C. Machado 958 (UEC 3ex); loc. cit., gruta linha 1, fl., fr., 27 Dec 1985, J.N.C. Marchiori 149 (HDCF).

##### Distribution and habitat.

*Tradescantia valida* is endemic to Brazil, more precisely to the state of Rio Grande do Sul (Fig. 8). It can be found growing as rupicolous, rarely as a terrestrial, in rocky walls.

##### Phenology.

It was found in bloom and fruit in December and April.

##### Conservation status.

*Tradescantia valida* is only known from the cultivated type collection and collections in Jaguari, state of Rio Grande do Sul. Thus, in accordance with the IUCN recommendations (IUCN 2001), it should be considered as Data Deficient (DD), until further information on the species becomes available.

##### Discussion.

*Tradescantia valida* is closely related to the remaining three species in the *T. crassula* group, due to its erect habit, definite base, sessile, conduplicate to falcate, succulent leaves, generally with inconspicuous secondary veins, cincinni bracts non-saccate at base, pistil longer than the stamens, hilum longer than ½ the length of the seed, and for preferentially inhabiting open areas and rocky outcrops (Pellegrini 2015, 2016). *Tradescantia valida* can be differentiated easily from all remaining species of the *T. crassula* group by its spathaceous cincinni bracts and by the presence of supernumerary bracts. The presence of spathaceous cincinni bracts is a character previously reported in T. sect. Austrotradescentia exclusively for *T. umbraculifera* Hand.-Mazz., a member of a clade named *T. fluminensis* group by Pellegrini (2015), and thus quite remarkable in this distantly related species. Aside from that, the presence of supernumerary bracts is unique within T. sect. Austrotradescantia, but well-known in species from T. sect. Cymbispatha (Pellegrini 2015; Pellegrini et al. 2016) and T. sect. Mandonia (Grant 2000; Pellegrini 2015).

In the *T. crassula* group, *T. valida* is similar to *T. cerinthoides* due to its sepals without dorsal keels. However, they can be easily differentiated due to its generally linear elliptic to linear lanceolate to lanceolate leaf-blades (*vs.* elliptic to broadly elliptic or ovate to broadly ovate or obovate to broadly obovate, in *T. cerinthoides*), glabrous with margins setose at the base or until the middle (*vs.* pubescent on both sides or only abaxially, rarely glabrous on both sides with ciliate margins), and pedicels and sepals glabrous or only sparsely pubescent with eglandular hairs (*vs.* evenly densely velutine to hispid, sometimes with a mixture of glandular and eglandular hairs). *Tradescantia valida* is much more similar to *T. crassula* and *T. seubertiana* M.Pell., due to their leaf-blades and floral pubescence. These species can be easily differentiated by the pubescence of the margin of their leaf-sheaths (ciliate to shortly-setose in *T. crassula*; glabrous in *T. seubertiana*; and long-setose in *T. valida*), the pubescence of their sepals (long-setose along the keels in *T. crassula*; glabrous in *T. seubertiana*; and glabrous or with few hairs at the apex in *T. valida*), and by the shape of their floral buds (broadly ovoid *T. crassula*; ellipsoid in *T. seubertiana*; and ellipsoid in *T. valida*).

### Updated key to the *Tradescantia crassula* group (modified from Pellegrini 2016)

**Table:** 

1	Leaf-blades pubescent on both sides or only abaxially, rarely glabrous on both sides; pedicels and sepals densely velutine to hispid, sometimes with a mixture of glandular and eglandular hairs	***Tradescantia cerinthoides* Kunth**
–	Leaf-blades glabrous on both sides, secondary veins adaxially inconspicuous; pedicels glabrous, rarely sparsely glandular-pubescent, sepals glabrous or with hairs restricted to the dorsal keel	**2**
2	Leaf-blades with margins setose at base or until the middle with long hairs; supernumerary bracts present, cincinni bracts spathaceous; sepals not dorsally keeled	***Tradescantia valida* G.Brückn.**
–	Leaf-blades with margin entire or ciliolate to ciliate; supernumerary bracts absent, cincinni bracts leaf-like; sepals dorsally keeled	**3**
3	Leaf-sheaths margins glabrous, base of the blades cordate to slightly amplexicaulous to obtuse; cincinni bracts unequal; floral buds ellipsoid; sepals glabrous; petals light pink to pink	***Tradescantia seubertiana* M.Pell.**
–	Leaf-sheaths margins ciliolate to ciliate, base of the blades obtuse to truncate; cincinni bracts equal; floral buds broadly ovoid; sepals sparsely setose along the keel; petals white	***Tradescantia crassula* Link & Otto**

#### Tradescantia sect. Campelia

Taxon classificationPlantaeCommelinalesCommelinaceae

(Rich.) D.R.Hunt, Kew Bull. 41(2): 404. 1986.

Fig. 4

##### Diagnosis.

The section is characterized by perennial herbs, with thin fibrous roots, definite base, without rhizomes, leaves with symmetric or slightly asymmetric base, inflorescences axillary, pedunculate, cincinni bracts spathaceous, bracteoles conspicuous and linear, flowers tubular, sepals unequal, basally conate, not keeled, petals free, shortly-clawed, stamens 6 and subequal, free, filaments straight at post anthesis, medially sparsely bearded with moniliform hairs, connectives sagittate, anther sacs round, ovary glabrous, stigma capitate, seeds smooth to faintly rugose, embryotega inconspicuous and semilateral (Hunt 1986; Pellegrini 2015).

**Figure 4.**
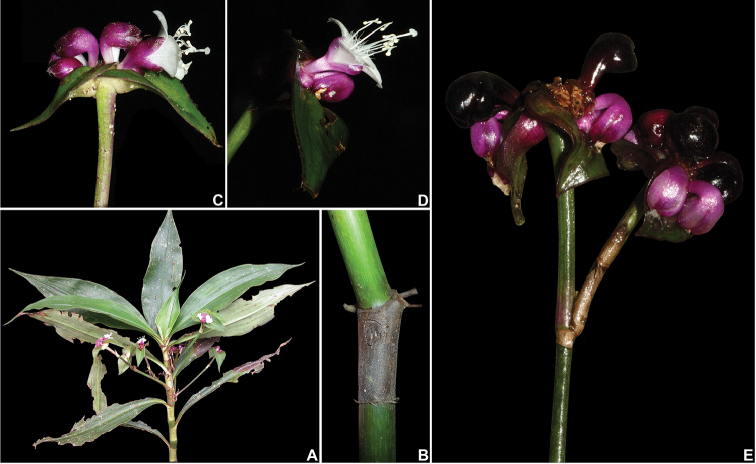
*Tradescantia zanonia* (L.) Sw. **A** habit, showing the axillary inflorescences perforating the leaf-sheaths **B** detail of the leaf-sheath **C** detail of the inflorescence, showing the spathaceous, saccate cincinni bracts, geniculate flowers, and basally conate sepals **D** side view of a flower, showing the shallowly-tubular flower, subequal stamens, sagittate connectives, round anther sacs, and trilobate stigma **E** detail of a branched synflorescence, bearing berry-like fruits. Photograph A by A.P. Maceda, B by P. Schwirkowski, C–E by M.O.O. Pellegrini.

##### Comments.

Tradescantia sect. Campelia is monospecific and represented by *T. zanonia* (L.) Sw. It was considered by Hunt (1986) to be unique within the genus due to its fleshy pedicel and sepals covering the capsule, giving the fruit a berry-like appearance, which is consumed by birds and other small animals (Hunt 1986; Pellegrini, pers. obs.). Nonetheless, the morphological similarity to Tradescantia sect. Zebrina is indisputable, as pointed out by Hunt (1986) and here reaffirmed by us. Tradescantia sect. Zebrina is highly variable in the following characters: (1) the position of the inflorescence; (2) if it perforates the leaf-sheaths or not; (3) the degree of conation between the sepals, the petals; and (4) the degree of connation between the petals and stamens. Thus, both sections are distinguished solely based on the consistency of their calyx (Pellegrini, pers. obs.).

#### Tradescantia sect. Mandonia

Taxon classificationPlantaeCommelinalesCommelinaceae

D.R.Hunt, Kew Bull. 35(2): 441. 1980.

##### Diagnosis.

The section is characterized by perennial herbs, with tuberous roots, definite base, without rhizomes, leaves with symmetric or slightly asymmetric base, inflorescences mainly axillary, sessile, cincinni bracts much reduced or rarely leaf-like in the terminal inflorescences, bracteoles inconspicuous, flowers flat, rarely tubular, sepals equal, free, not keeled, petals free, sessile, stamens 6 and equal, free or epipetalous, filaments coiling at post anthesis, medially sparsely bearded with moniliform hairs, connectives quadrangular to rectangular, rarely rhomboid, anther sacs C-shaped, ovary pubescent, stigma truncate to capitulate, seeds scrobiculate to rugose, rarely costate, embryotega conspicuous and dorsal (Hunt 1980; Pellegrini 2015).

##### Comments.

Tradescantia sect. Mandonia is a poorly understood group, currently represented by 12 species (Hunt 1980, 1986b, 2007; Grant 2000, 2004; Zamudio et al. 2013). This section possesses a very interesting disjunctive distribution, being restricted to Seasonally Dry Tropical Forests throughout the Neotropics. Species delimitation is especially complicated in this this group, due to great vegetative plasticity within species, and conserved reproductive features. Most species can be easily identified based on their allopatric distributions, but hardly differentiated based solely on their morphological features (Pellegrini, unpublished data). In South America, T. sect. Mandonia is currently represented exclusively by two species, *T. ambigua* Mart. *ex* Schult. & Schult.f. and *T. boliviana*. *Tradescantia boliviana* has hitherto been considered exclusive to Argentina, Bolivia, Paraguay, and Peru (Grant 2004). However, after analyzing several herbaria we came across specimens representing *T. boliviana* that reached the Brazilian territory, in the state of Mato Grosso do Sul. Thus, we present an identification key differentiating both species, illustrations, a distribution map, and the needed comments and typifications.

### Key to the South American species of Tradescantia sect. Mandonia

**Table:** 

1	Leaves flat, abaxially hispid; pedicel and sepals velutine, connective rectangular, ovary and capsules densely velutine to velutine; hilum ½ the length of the seed	***Tradescantia ambigua* Mart. *ex* Schult. & Schult.f.**
–	Leaves conduplicate, abaxially densely velutine; pedicel and sepals hispid or glandular-pubescent, connective quadrangular, ovary and capsules velutine to sparsely velutine at apex; hilum as long as the seed	***Tradescantia boliviana* (Hassk.) J.R.Grant**

#### Tradescantia ambigua

Taxon classificationPlantaeCommelinalesCommelinaceae

Mart. ex Schult. & Schult.f. in Roemer & Schultes, Syst. Veg. (ed. 15 bis) 7(2): 1170. 1830.

Figs 5 , 8

Tradescantia ambigua Lectotype (designated here). BRAZIL. Bahia: Provincia Bahiensis, fl., fr., s.dat., C.F.P. von Martius 140 (M barcode M0243603!).
Tradescantia ambigua var. glabriuscula C.B.Clarke, Monogr. Phan. 3: 292. 1881. Lectotype (designated here). BRAZIL. Piauhy, shady woods between São Gonçalo do Piauí and Campos, fl., Feb 1819, G. Gardner 2334 (K barcode K000363268!; isolectotype: BM barcode BM001209590!).

##### Diagnosis.

*Herbs* perennial, with a definite base, terrestrial to rupicolous. *Roots* thick, tuberous, brown to dark-brown, densely to sparsely pilose with brown to dark-brown hairs emerging from the rhizome and from the basal-most nodes. *Stems* erect, succulent, unbranched, rarely branched near the base; internodes green to vinaceous to reddish brown, sometimes with green striations, sparsely velutine, becoming glabrous at age, hairs hyaline. *Leaves* spirally-alternate, evenly distributed along the stems, sessile, the apical ones gradually smaller than the basal ones; sheaths green, hispid, hairs hyaline, margins hispid, hairs hyaline; lamina crass, flat, light to medium green on both sides, lanceolate to ovate, rarely obovate, adaxially sparsely hispid, becoming glabrous when mature, abaxially densely hispid, hairs hyaline, base truncate to amplexicaulous, margins vinaceous to reddish brown, ciliate, apex acuminate; midvein conspicuous, impressed adaxially, prominently obtuse abaxially, secondary veins inconspicuous to slightly conspicuous on both sides. *Inflorescences* (*main florescences*) consisting of a sessile double-cincinni fused back to back, axillary in the uppermost nodes; peduncles inconspicuous; basal bract inconspicuous, tubular, hyaline, glabrous; peduncle bracts absent; supernumerary bracts sometimes present, 1–3 per inflorescence, similar in shape and size to the cincinni bracts; cincinni bracts reduced, light green, hispid, base non-saccate; cincinni 6–12-flowered; bracteoles inconspicuous, imbricate, linear-triangular to triangular, hyaline. *Flowers* bisexual, actinomorphic, flat (not forming a floral tube); floral buds ovoid, light to medium green, rarely pink; pedicels light to medium green, rarely pink, velutine to hispid, hairs eglandular, hyaline; sepals 3, equal, ovate, cucullate, dorsally not keeled, light to medium green, rarely pink, velutine to hispid, hairs eglandular, hyaline, apex acute, margins hyaline light-green, persistent in fruit; petals 3, equal, sessile, elliptic to ovate to rhomboid, white to pale lilac to lilac to light pink to pink, base cuneate, margins entire, apex acute; stamens 6, equal, filaments light pink to pink to dark pink, rarely white, medially densely bearded with moniliform, light pink to pink to dark pink hairs, straight at anthesis, coiling at post anthesis, connective expanded, rectangular, yellow to orange, anthers sacs curved, yellow, pollen yellow; ovary subglobose, 3-loculate, white, smooth, densely velutine, style straight, white to light pink to pink, stigma capitulate, white to light pink, pistil longer than the stamens. *Capsules* subglobose, apiculate due to persistent style apex, light green when immature, light brown when mature, velutine, smooth, 3-valved. *Seeds* uniseriate, 2 per locule, ellipsoid to broadly ellipsoid, not cleft towards the embryotega, ventrally flattened, testa grey to greyish brown, farinose, scrobiculate arranged in ridges radiating from the embryotega; embryotega dorsal, conspicuous, with a prominent apicule; hilum linear, ½ the length of the seed.

**Figure 5.**
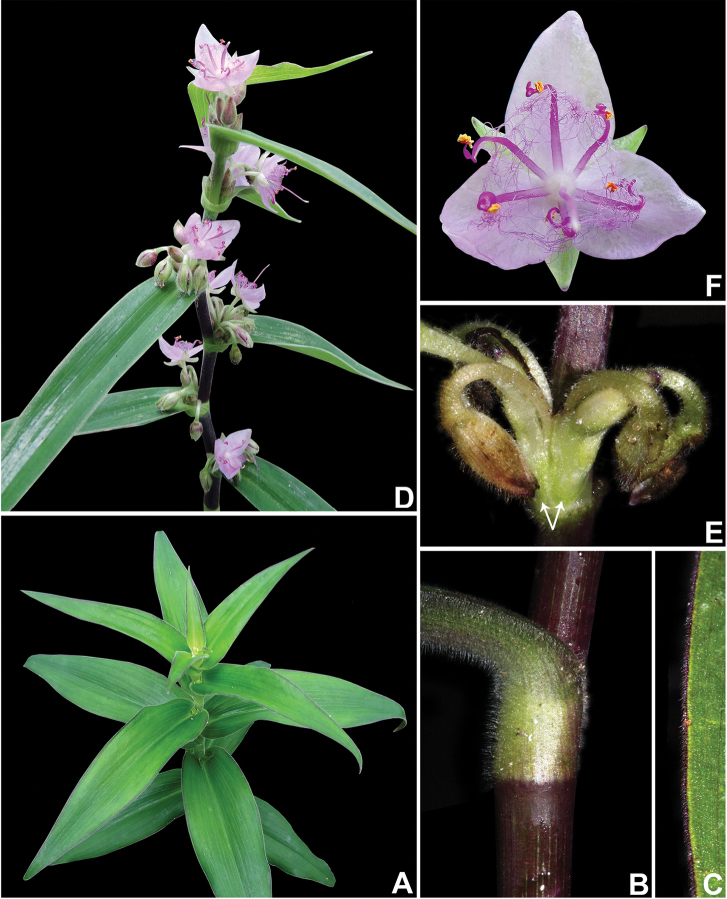
*Tradescantia ambigua* Mart. **A** sterile habit, showing the amplexicaulous leaf-blades **B** detail of the stem and leaf-sheath, showing the densely hispid leaf-sheath and blade **C** detail of the leaf-blade, showing the reddish, ciliate margin, and the sparsely hispid blade **D** fertile habit, showing the axillary, sessile inflorescences **E** removed leaf, exposing the sessile inflorescence and showing the reduced cincinni bracts (arrows) **F** post-anthesis flower, showing the apically spirally-coiled filaments. Photograph A by E.O. Moura, D & F by L.J. Leitão, B–C & E by M.O.O. Pellegrini.

##### Specimens seen.

**BRAZIL. Alagoas**: Arapiraca, estrada em direção de Jaramataia, ca. 40km da cidade de Arapiraca, fl., 13 Jun 1980, V.C. Lima et al. 77 (IPA); Pão de Açúcar, caminho para Ilha do Ferro, fl., fr., 21 Jun 2002, R.P. Lyra-Lemos 6828 (HUEFS, MAC); Poço das Trincheira, Sítio Saco do Ramalho, fl., fr., 24 Aug 1983, M.N.R. Staviski et al. 643 (MAC, MG). **Bahia**: Cachoeira, Morro Belo, Vale dos Rios Paraguaçú e Jacuípe, fl., fr., Aug 1980, Grupo Scardino et al. 513 (CEPEC); Caculé, ca. 4.7 km E da sede municipal, estrada que leva à torre de televisão, fl., 30 Mar 2001, J.G. Jardim et al. 3213 (CEPEC, HUEFS, RB); Feira de Santana, distrito de Ipuaçu, fl., fr., 26 Jul 2003, F. França et al. 4798 (HUEFS, HRCB); loc. cit., fl., 19 May 2005, A.P.L. Couto et al. 96 (HUEFS); loc. cit., inselberg Monte Alto, fl., 19 Jun 2006, E. Melo & B.M. Silva 4453 (HUEFS); loc. cit., fl., fr., 8 Jul 2006, C.T. Lima et al. 39 (HUEFS); Itaberaba, Fazenda Morros, fl., fr., 15 Sep 1984, G. Hatschbach 48199 (MBM); Milagres, Morro Pé da Serra, fl., 16 Mar 1997, F. França et al. 2174 (HUEFS, UEC); Paulo Afonso, Raso da Catarina, Mata das Pororocas, fl., 10 Jun 1983, L.P. Queiroz 561 (ALCB, HUEFS, IPA); Riachão do Jacuípe, fl., fr., 6 Jun 2009, E. Melo et al. 6244 (HUEFS). **Ceará**: Aiuaba, Estação Ecológica de Aiuaba, Gameleira de Cima, fl., 9 Apr 1997, L.W. Lima-Verde et al. 570 (EAC); loc. cit., fl., 9 Apr 1997, L.W. Lima-Verde et al. 573 (EAC); Carnaubal, Planalto do Ibiapaba, fl., 30 Apr 2010, E.M. Marreira et al. 84 (HUEFS, HUVA); Crateús, Sertão de Crateús, Ibiapaba Norte, Picote, fl., fr., 21 May 1997, M.A. Figueiredo s.n. (EAC25685); General Sampaio, RPPN Fancy Nunes, fl., 25 May 2007, M.F. Moro et al. 137 (EAC); Ipueiras, Olho D’água dos Galvão, Nova Fátima, fl., fr., 20 Apr 2014, A.S.F. Castro 2802 (EAC); Itapagé, Serrote do Meio, Pitombeira, fl., 30 Mar 2002, A.S.F. Castro 1182 (EAC); Meruoca, Serra de Meruoca, Sítio Santo Antônio, fl., 15 Feb 1957, A. Fernandes s.n. (EAC1699); loc. cit., fl., 3 Mar 1962, A. Fernandes s.n. (EAC2158); Monsenhor Tabosa, Serra Branca/Serra das Matas, fl., 6 Mar 2000, A.S.F. Castro 804 (EAC); Novo Oriente, Baixa Fria, fl., fr., 4 May 1991, F.S. Araújo 445 (EAC); Pentecoste, Serra do Tamanduá, fl., 31 Mar 2001, A. Andrade s.n. (EAC30590); Santa Quitéria, Serra do Pajé, Fazenda Itan de Cima, fl., fr., 7 May 1997, L.W. Lima-Verde 733 (EAC); loc. cit., fl., fr., 7 May 1997, L.W. Lima-Verde 734 (EAC); loc. cit., Fazenda Itataia, fl., 24 Apr 1984, A. Fernandes et al. s.n. (EAC12489); loc. cit., Subida da Serra, cerca de 2.8km NE da Fazenda Itataia, fl., 27 Apr 2012, J. Paula-Souza et al. 11037 (EAC, ESA, RB); Tianguá, Chapada da Ibiapaba, Cachoeira do Frade, fl., fr., 30 Apr 1987, A. Fernandes & Matos s.n. (EAC15125, IPA69126). **Goiás**: Lavandeira, 5.5km de Lavandeira em direção a Aurora do Tocantins, fl., 25 Jan 2005, J. Paula-Souza et al. 4654 (ESA, RB). **Minas Gerais**: s.loc., s.dat., fl., A.F.M. Glaziou 14362 (US); Januária, Brejo do Amparo, Serra do Brejo, fl., 3 Jan 1970, J.P. Carauta 1058 (RB); loc. cit., 15 km na estrada a W de Januária para a Serra das Araras, fl., fr., 20 Apr 1973, W.R. Anderson et al. 9220 (MO, UB); loc. cit., Barreiro, estrada vicinal a partir do trevo do aeroporto, fl., 4 Apr 2016, C.N. Fraga 3654 (RB); Pains, st., 23 Jan 1991, M.A. Rollo s.n. (RB00898260, SPF). **Paraíba**: Pocinhos, Mubuco, fl., fr., 8 Jul 1994, A.M. Miranda & L.P. Félix 1844 (HST, PEUFR, RB, US); Remígio, Agreste, Escola Agronômica do Nordeste, fl., fr., 8 Aug 1958, J.C. Moraes 1854 (EAN, RB); São José dos Cordeiros, fl., 23 Mar 2003, I.B. Lima et al. 83 (HUEFS). **Pernambuco**: Alagoinha, Serra do Gavião, fl., fr., 8 Aug 2000, A. Viana et al. 75 (IPA); Arcoverde, Estação Experimental, fl., fr., 22 Jul 1971, Andrade-Lima 71-6399 (IPA); Betania, Serra dos Arrombadores, fl., 6 Apr 2002, S.M.F. Neto et al. 2 (IPA); Buique, Parque Nacional do Catimbau, perto da entrada da trilha do cânion, fl., fr., 16 May 2006, E.A. Rocha et al. 1481 (IPA); loc. cit., Vale do Catimbau, trilha das Torres, fl., fr., 18 Jun 2008, R. Pereira et al. 2760 (HUEFS, IPA); Pesqueira, Serra do Gavião, fl., fl., 19 Jun 2005, M. Oliveira 1811 (IPA); Ouricuri, margem da estrada Lagoa-Ouricuri, fl., 4 May 1971, E.P. Heringer et al. 499 (IPA, PEUFR, R, RB, UB); São Caetano, RPPN Pedra do Cachorro, Subida florestal do afloramento rochoso granítico, fl., fr., 19 Jun 2011, K. Mendes, 700 (ASE). **Rio Grande do Norte**: Bodó, fl., 4 May 2014, E.O. Moura et al. 161 (UFRN); Florânia, rodovia para Tenente Laurentino ca. 6 km da sede municipal, Serra de Santana, fl., 29 May 2010, J.G. Jardim et al. 5768 (RB, UFRN); Serra de São Bento, fl., L.M. Versieux et al. 548 (HURB, RB, UFRN). **Sergipe**: Canindé de São Francisco, fl., 1 Sep 2014, G.S. Freire 116 (ASE); Frei Paulo, 6km após o povoado Mocambo, entrada para mata, fl., 26 Jun 1981, M. Fonseca 517 (ASE); loc. cit., fl., fr., 6 Aug 1987, G. Viana 1925 (ASE, HURB); Nossa Senhora da Glória, Fazenda Olhos d’Água, fl., fr., 06 Aug 1982, E. Gomes 114 (ASE); loc. cit., fl., fr., 1 Sep 1983, G. Viana 765 (ASE, HURB); loc. cit., fl., 6 May 1986, G. Viana 1456 (ASE); Porto da Folha, povoado Lagoa do Rancho, fl., fr., 20 Jul 2006, E. Córdula et al. 115 (HUEFS, UFP); loc. cit., Fazenda São Pedro, fl., 19 Apr 2011, D.G. Oliveira 150 (ASE, MAC); loc. cit., fl., fr., 6 Aug 2012, A.P. Prata at al. 3212 (ASE); loc. cit, fl., fr., 5 Aug 2014, L.A.S. Santos 1158 (ASE, RB). **Without state**: s.loc., fl., M.A. Glaziou 14362 (P, US).

##### Distribution and habitat.

*Tradescantia ambigua* is endemic to Northeastern Brazil (states of Alagoas, Bahia, Ceará, Paraíba, Pernambuco, Piauí, Rio Grande do Norte, and Sergipe), also reaching the states of Goiás and Minas Gerais. It grows in moist and shady areas, between shrubs and patchy forests in the Caatinga and Cerrado domains (Fig. 8).

##### Phenology.

It was found in bloom and fruit from January to August.

##### Conservation status.

*Tradescantia ambigua* possesses a wide EOO (ca. 1,057,693.924 km^2^), and following the IUCN recommendations (IUCN 2001), it should be considered Least Concern (LC). However, when taking the AOO into consideration, it is considerably reduced (ca. 216.000 km^2^). Furthermore, The Caatinga and Cerrado domains are greatly threatened by human activities such as deforestation, cattle breeding, and various crops. Thus, following the IUCN (2001) recommendations, *T. ambigua* should be considered Endangered [EN, A2c+B2ab(ii, iii, iv, v)+C2a(i)].

##### Nomenclatural notes.

When describing *T. ambigua*, Schultes and Schultes (1830) mention that their new species is based on a Martius specimen from the Province of Bahia, but with no reference to herbaria or collector number. While consulting M we came across a specimen (*Martius 140*) matching perfectly the protologue, and annotated in Martius’s handwriting. Thus, it is here designated as the lectotype.

When describing T. ambigua var. glabriuscula, Clarke (1881) only lists the collection *Gardner 2334*. However, Clarke makes no reference to the herbaria in which this specimen might be distributed. After analyzing several collections, we found specimens of *Gardner* 2334 at BM and K. According to Stafleu and Cowan (1976), Clarke had access to both collections, and many of the type specimens for names described by him were commonly from either of the two herbaria. The specimen at K is composed of a much longer stem, containing a greater number of leaves, inflorescences and flowers. Added to that, it is annotated on Clarke’s handwriting, making it the obvious choice for a lectotype.

##### Morphological notes.

*Tradescantia ambigua*, as most species in T. sect. Mandonia, presents a high degree of morphological variation. The plants vary greatly in size, branching of the stem, shape of the leaves, pubescence of the leaves, pubescence of the pedicels and sepals, and shape and color of the petals. Nonetheless, this variation has no obvious geographical pattern and seems rather random across its distribution range. Thus, don’t recognize any infraspecific taxa for *T. ambigua*.

#### Tradescantia boliviana

Taxon classificationPlantaeCommelinalesCommelinaceae

(Hassk.) J.R.Grant, Novon 14(3): 299. 2004.

Figs 6 , 8

Skofitzia boliviana (Hassk.) Hassk. & Kanitz, Oesterr. Bot. Z. 22: 147. 1872.
Mandonia boliviana Hassk., Flora 54: 260. 1871. Lectotype (designated by Grant 2004). BOLIVIA. Larecaja: viciniis Sorata montis colles Ullontigi ad scopulorumra dicemi n regionet emperata, fl., fr., Feb–Apr 1858, G. Mandon 1239 (L barcode L0374955!; isolectotypes: F barcode F0076407F!, G barcode G00489633!, GH n.v., K n.v., P 3ex barcodes P00376711!, P00376712!, P00376713!).
Tradescantia ambigua var. pilosula Hoehne, Relat. Commiss. Linhas. Telegr. Estrateg. Matto Grosso Amazonas 5, 5: 14. 1915. Lectotype (designated here). BRAZIL. Mato Grosso do Sul: Corumbá, fl., Feb 1911, F.C. Hoehne 4499 (R barcode R000004848!). **Syn. nov.**

##### Diagnosis.

*Herbs* perennial, with a definite base, terrestrial to rupicolous. *Roots* thick, tuberous, brown to dark-brown, densely to sparsely pilose with brown to dark-brown hairs, emerging from the rhizome and from the basal most nodes. *Stems* erect, succulent, unbranched, rarely branched near the base; internodes green to vinaceous to reddish brown, sometimes with vinaceous striations, densely velutine, sometimes becoming glabrous at age, hairs hyaline to light brown. *Leaves* spirally-alternate, evenly distributed along the stems, sessile, the apical ones gradually smaller than the basal ones; sheaths green, densely velutine, hairs hyaline to light brown, margins ciliate, hairs hyaline to light brown; lamina succulent conduplicate, medium to dark green adaxially, light to medium green abaxially, narrowly lanceolate to lanceolate, rarely ovate, adaxially sparsely velutine, sometimes becoming glabrous at age, abaxially densely velutine, hairs light brown, base truncate to rounded, margins vinaceous to reddish brown, ciliate, apex acute; midvein conspicuous, impressed adaxially, prominently obtuse abaxially, secondary veins inconspicuous to slightly conspicuous on both sides. *Inflorescences* (*main florescences*) consisting of a sessile double-cincinni fused back to back, axillary in the uppermost nodes; peduncles inconspicuous; basal bract inconspicuous, tubular, hyaline, glabrous; peduncle bracts absent; supernumerary bracts sometimes present, 1–3 per inflorescence, similar in shape and size to the cincinni bracts; cincinni bracts reduced, light green, hispid, base non-saccate; cincinni (4–)8–22-flowered; bracteoles inconspicuous, imbricate, linear-triangular to triangular, hyaline. *Flowers* bisexual, actinomorphic, flat (not forming a floral tube); floral buds narrowly ovoid, dark pink to vinaceous; pedicels dark pink to vinaceous, densely glandular-pubescent, hairs light brown; sepals 3, equal, ovate, cucullate, dorsally not keeled, dark pink to vinaceous, rarely green, densely glandular-pubescent, hairs light brown, apex acute, margins hyaline light-green, persistent in fruit; petals 3, equal, sessile, broadly ovate, medium to dark pink to mauve, rarely white or light pink to lilac, base cuneate, margins entire, apex acute; stamens 6, equal, filaments light medium to dark pink to mauve, medially bearded with moniliform, medium to dark pink to mauve hairs, straight at anthesis, coiling at post anthesis, connective expanded, quadrangular, yellow to orange, anthers sacs curved, yellow, pollen yellow; ovary oblongoid, 3-loculate, white, smooth, velutine at apex, style straight, medium to dark pink, stigma capitulate, white to light pink, pistil longer than the stamens. *Capsules* broadly oblongoid, apiculate due to persistent style apex, green when immature, brown when mature, sparsely velutine at the apex, smooth, 3-valved. *Seeds* uniseriate, 2 per locule, ellipsoid to broadly ellipsoid, not cleft towards the embryotega, ventrally flattened, testa grey to greyish brown, farinose, scrobiculate arranged in ridges radiating from the embryotega; embryotega dorsal, conspicuous, with a prominent apicule; hilum linear, as long as the seed.

**Figure 6.**
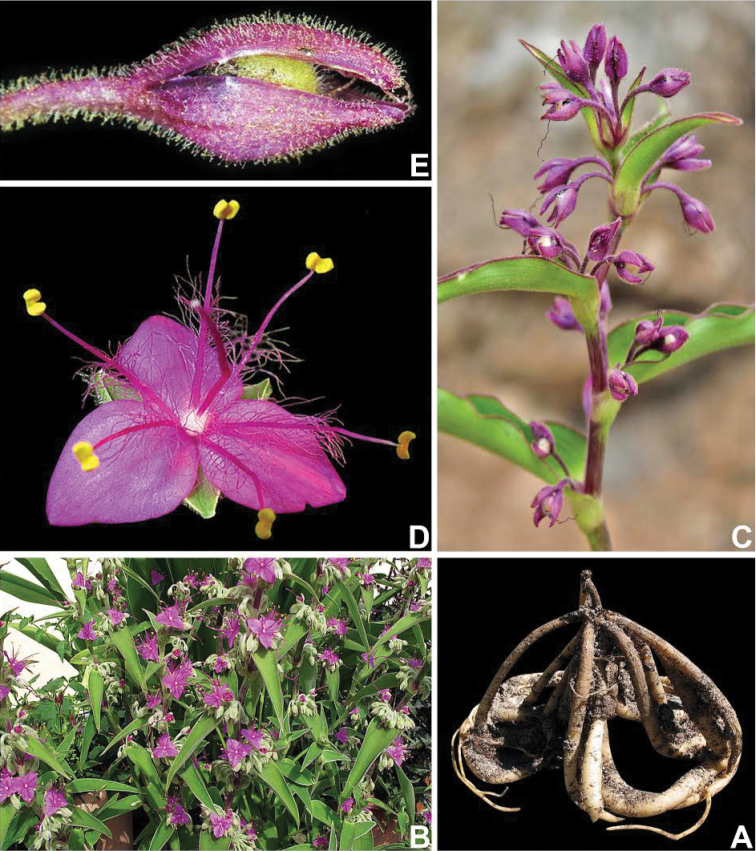
*Tradescantia boliviana* (Hassk.) J.R.Grant. **A** detail of the tuberous roots **B** habit **C** detail of fertile branch, showing the conduplicate leaf-blades, and axillary inflorescences **D** front view of a flower at anthesis, showing the rectangular connectives and C-shaped anther sacs **E** detail of an immature capsule, showing the densely glandular-pubescent pedicel and sepals, and the capsule apically velutine. Photograph A–B & D by P. Christian (RarePlants.co.uk), C & E by Instituto Darwinion.

##### Specimens seen.

**BRAZIL. Mato Grosso do Sul**: Campo Grande, Empraba Gado de Corte, fl., fr., 28 Feb 2009, V.J. Pott 10452 (CGMS, HURB); Corumbá, fl., Feb 1911, F.C. Hoehne 4723 (R); loc. cit., Mineração Corumbaense Reunida SA, paredão próximo ao paiol de esplosivos, fl., fr., 23 Feb 2005, G.A. Damasceno-Junior et al. 3488 (COR, K); loc. cit., Serra Urucum, fl., fr., 24 Feb 2005, A. Pott et al. 12655 (CGMS, HMS); loc. cit, Fazenda Banda Alta, fl., 11 Jan 2007, A. Takahasi & S.M. Ribas 1175 (COR); Ladário, Fazenda São Sebastião do Carandá, fl., fr., 13 Mar 2003, G.A. Damasceno-Junior et al. 2773 (COR); Nioaque, Assentamento Andalucia, fl., 6 Dec 2008, L.C.S. Magalhães & G.A. Damasceno-Júnior 138 (CGMS, HURB); loc. cit., fl., fr., 9 Jan 2009, L.C.S. Magalhães & T.S. Amaral 194 (CGMS, HURB); loc. cit., fl., fr., 9 Jan 2009, L.C.S. Magalhães & T.S. Amaral 195 (CGMS); loc. cit., fl., fr., 8 Feb 2009, L.C.S. Magalhães & T.S. Yule 278 (CGMS).

##### Distribution and habitat.

*Tradescantia boliviana* restricted to Argentina, Bolivia, Paraguay, Peru, and Brazil (state of Mato Grosso do Sul). It commonly grows as rupicolous in rocky walls and outcrops, under full sunlight, in the Chaco and Pantanal domains (Fig. 8).

##### Phenology.

It was found in bloom and in fruit from December to June.

##### Conservation status.

*Tradescantia boliviana* possesses a wide EOO (ca. 2,249,457.700 km^2^), and based solely on this criterion it should be considered Least Concern (LC). Nonetheless, its AOO is considerably reduced (ca. 172.000 km^2^), added to that fact that most of the studied specimens are at least more than 20 years old. Thus, following the IUCN (2001) recommendations, *T. boliviana* should be considered Endangered [EN, A2bcd+B2ab(ii, iii, iv, v)+C1+C2a(i)].

##### Nomenclatural notes.

In the protologue of T. ambigua var. pilosula, Hoehne (1915) mentions two collections of his own when describing this new variety. The author makes no reference as to the herbarium in which each specimen is placed or to the existence of possible duplicates. According to Stafleu and Cowan (1979), Hoehne’s types are generally at SP with duplicates in several other herbaria. However, after two visits to the SP we were unable to locate any of the specimens (*Hoehne 4499*, *4723*). Nonetheless, Stafleu and Cowan (1979) make an important remark that until 1917, Hoehne was living and working in Rio de Janeiro. After analyzing the collection of R, we came across both specimens placed in the general collection. Both specimens were annotated by Hoehne, but the specimen Hoehne 4499 possesses a beautiful illustration attached to it, showing the details of the plants’ floral morphology. Thus, it is designated by us as the lectotype for T. ambigua var. pilosula.

##### Morphological notes.

*Tradescantia boliviana* is a morphologically variable species across its distribution. Nonetheless, in the same way as *T. ambigua*, there is no obvious geographical pattern in this variation. The presence of glandular hairs in the pedicels and sepals can be observed in some of the individuals, but aside from that they don’t seem to differ in any other aspect from the other specimens. This variation is peculiar, but not unrecorded in the genus, and a similar scenario is described by Pellegrini (2015, 2016) for *T. cerinthoides* (T. sect. Austrotradescantia) and by Faden (1993) for *T. crassifolia* (T. sect. Mandonia).

After analyzing the type specimens for T. ambigua var. pilosula, we noticed that the pedicels and sepals are hispid, the connectives are quadrangular, and the ovary velutine to sparsely velutine at apex. Added to that, the distribution of the specimens collected by Hoehne is congruent with the distribution of *T. boliviana*, but disjunctive from *T. ambigua*. Thus, we consider T. ambigua var. pilosula a synonym of *T. boliviana*.

#### Tradescantia sect. Zebrina

Taxon classificationPlantaeCommelinalesCommelinaceae

(Schnizl.) D.R.Hunt, Kew Bull. 41(2): 404. 1986.

Fig. 7

##### Diagnosis.

The section is characterized by perennial herbs, with thin fibrous roots, definite or indefinite base, without rhizomes, leaves with symmetric to asymmetric base, inflorescences terminal or axillary, pedunculate, cincinni bracts spathaceous, bracteoles conspicuous and linear, flowers tubular, sepals unequal, basely to completely conate, keeled or not, petals free or conate, long-clawed, stamens 6 and subequal, epipetalous, filaments straight at post anthesis, medially sparsely bearded with moniliform hairs, connectives sagittate to linearly-tapered, anther sacs round, ovary glabrous, stigma capitate, seeds rugose, embryotega inconspicuous and semilateral (Hunt 1986; Pellegrini 2015).

**Figure 7.**
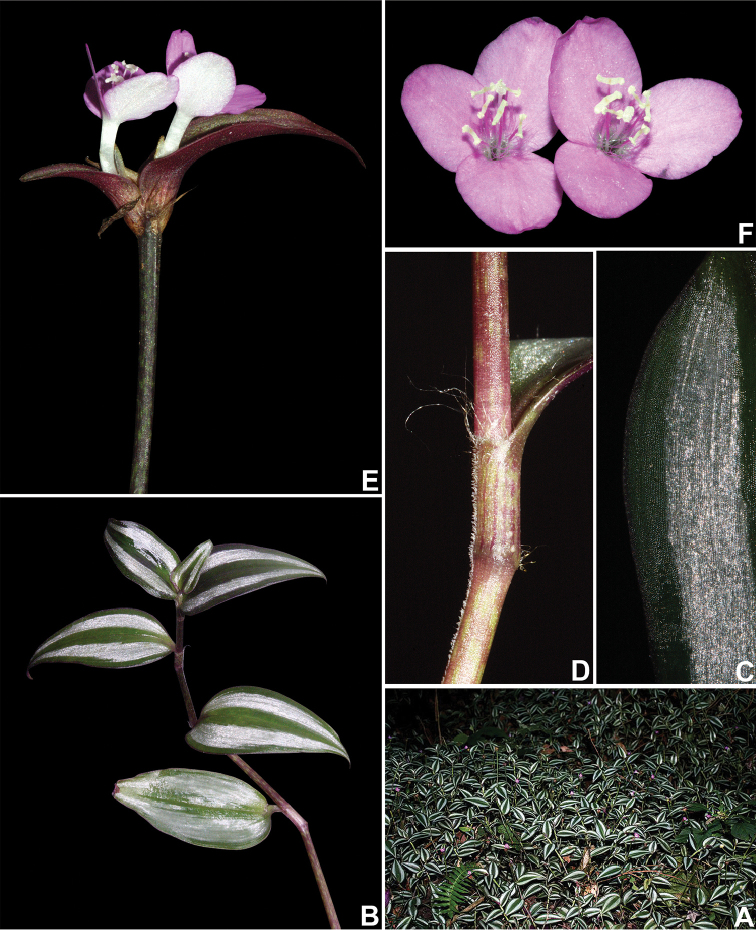
*Tradescantia zebrina* Heynh. *ex* Bosse. **A** habit **B** detail of a branch, showing the subpetiolate basal leaves, and blades with silver stripes **C** detail of the abaxial side of the leaf-blade **D** detail of the stem and leaf-sheath **E** detail of the terminal inflorescence, showing the spathaceous, saccate, unequal, conduplicate cincinni bracts, and long-tubular flowers with clawed petals **F** flowers. Photographs by M.O.O. Pellegrini.

##### Comments.

Tradescantia sect. Zebrina is a small group, composed of ca. five species, ranging from Mexico to Venezuela. *Tradescantia zebrina* Heynh. *ex* Bosse is widely cultivated worldwide, and occurs in Brazil as an invasive species (Hunt 1986; Pellegrini 2017). As aforementioned, the section is small but morphologically diverse, being poorly differentiated from T. sect. Campelia and T. sect. Corinna. As stated by Hunt (1986), these three sections seem to blur into one another, with several species being originally assigned to one group and subsequently transferred to another.

## Conclusion

*Tradescantia*, like many other genera in Commelinaceae, is a taxonomically complicated and morphologically diverse genus. In order to safely propose taxonomic novelties, it is necessary to possesses a broader knowledge on the group, especially regarding the morphological plasticity within each species. This can only be achieved with extensive field and herbaria research, complemented with the cultivation and observation of some individuals. Many recent studies of Brazilian Commelinaceae have been narrowly focused, and proposed new species and several typifications (Funez et al. 2016; Hassemer et al. 2016a, 2016b; Hassemer 2017). As demonstrated by us in the present work, this can lead to the unnecessary description of new names, causing the inflation of accepted species and their conservation assessments. Perhaps the most unfortunate result of such studies is the potential for incorrect typification and application of names (e.g. Funez et al. 2016; Hassemer et al. 2016b; Hassemer 2017). Thus, we strongly suggest that future typifications and descriptions of new taxa in Commelinaceae be carried out as part of a broader and more detailed taxonomic framework.

**Figure 8.**
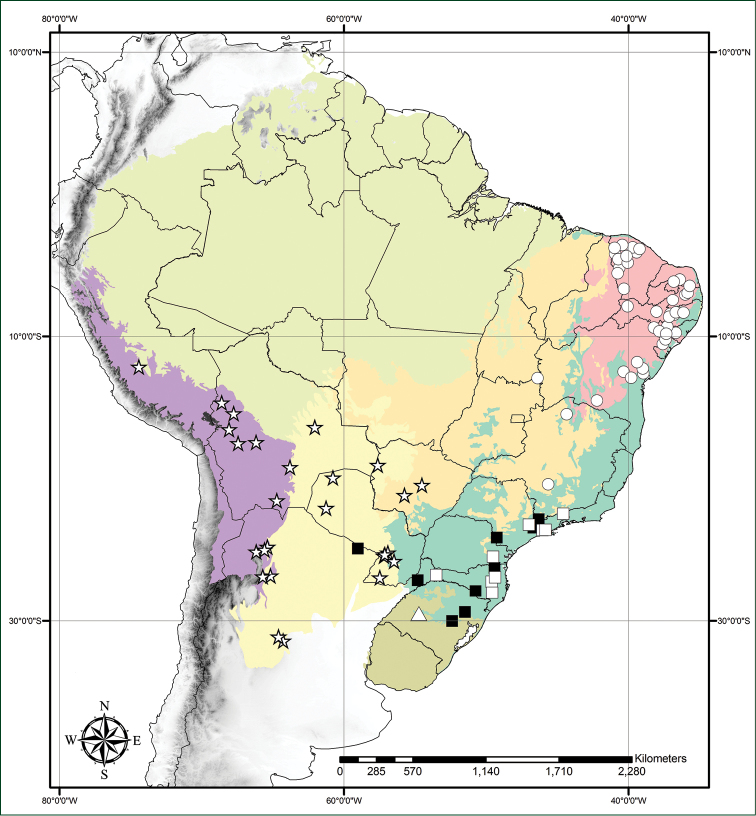
Distribution map of studied *Tradescantia* L. in the South American domains. **Full squares**– *T. crassula*; **White squares**– *T. chrysophylla*; **Triangles**– *T. valida*; **Circles**– *T. ambigua*; **Stars**– *T. boliviana*. Light green– Amazon Forest; Orange– Cerrado; Red– Caatinga; Yellow– Chaco and Pantanal; Olive-green– Pampa; Dark green– Atlantic Forest; Purple– Andean Yungas.

## Supplementary Material

XML Treatment for Tradescantia sect. Austrotradescantia

XML Treatment for Tradescantia crassula

XML Treatment for Tradescantia chrysophylla

XML Treatment for Tradescantia valida

XML Treatment for Tradescantia sect. Campelia

XML Treatment for Tradescantia sect. Mandonia

XML Treatment for Tradescantia ambigua

XML Treatment for Tradescantia boliviana

XML Treatment for Tradescantia sect. Zebrina

## References

[B1] Anderson ES Woodson RE Jr (1935) The species of *Tradescantia* indigenous to the United States. Contributions, Arnold Arboretum 9: 1–132.

[B2] Bachman S Moat J Hill AW Torre J Scott B (2011) Supporting Red List threat assessments with GeoCAT: geospatial conservation assessment tool. In: Smith V Penev L (Eds) e-Infrastructures for data publishing in biodiversity science. ZooKeys 150: 117–126. doi: 10.3897/zookeys.150.2109 10.3897/zookeys.150.2109 PMC3234434 22207809

[B3] Burns JH Faden RB Steppan SJ (2011) Phylogenetic studies in the Commelinaceae subfamily Commelinoideae inferred from nuclear ribosomal and chloroplast DNA sequences. Systematic Botany 36(2): 268–276. https://doi.org/10.1600/036364411X569471

[B4] Clarke CB (1881) Commelinaceae. In: De Candolle A (Ed.) Monographiae Phanerogamarum, vol. 3 Sumptibus G. Masson, Paris, France, 113–324, t. I–VIII.

[B5] eMonocot (2010) eMonocot, Version 1.0.2. http://e-monocot.org/ [accessed: 1.20.2017]

[B6] Evans TM Sytsma KJ Faden RB Givnish TJ (2003) Phylogenetic relationships in the Commelinaceae: II. A cladistic analysis of rbcL sequences and morphology. Systematic Botany 28: 270–292.

[B7] Faden RB (1991) The morphology and taxonomy of *Aneilema* R.Brown (Commelinaceae). Smithsonian Contributions to Botany (Washington, DC) 76: 1–181. https://doi.org/10.5479/si.0081024X.76

[B8] Faden RB (1993) *Tradescantia crassifolia* (Commelinaceae), an overlooked species in the southwestern United States. Ann. Missouri Bot. Gard. 80(1): 219–222. https://doi.org/10.2307/2399825

[B9] Faden RB (1998) Commelinaceae. In: Kubitzki K (Ed.) The Families and Genera of Vascular Plants (Vol. 4). Springer Verlag, Berlin, 109–128. https://doi.org/10.1007/978-3-662-03531-3_12

[B10] Faden RB Hunt DR (1991) The Classification of the Commelinaceae. Taxon 40(1): 19–31. https://doi.org/10.2307/1222918

[B11] Funez LA Hassemer G Ferreira JPR (2016) Description of *Tradescantia schwirkowskiana* (Commelinaceae), a narrow endemic new species from Santa Catarina, Southern Brazil, and typification of *T. crassula*. Phytotaxa 272(1): 63–72. https://doi.org/10.11646/phytotaxa.272.1.3

[B12] Grant JR (2000) New Mesoamerican species of *Dichorisandra* and Tradescantia section Mandonia (Commelinaceae). Novon 10(2): 117–123. https://doi.org/10.2307/3393009

[B13] Grant JR (2004) *Tradescantia boliviana* (Commelinaceae), a new combination for an overlooked South American species. Novon 14(3): 299–300.

[B14] Hassemer G Ferreira JPR Funez LA Aona LYS (2016) Identity and typification of *Commelina vilavelhensis* (Commelinaceae), and typification of *C. robusta* and *C. scabrata*. Phytotaxa 260(2): 144–156. https://doi.org/10.11646/phytotaxa.260.2.4

[B15] Hassemer G Ferreira JPR Funez LA Medeiros JDM (2016) *Commelina catharinensis* (Commelinaceae): a narrow endemic and endangered new species from Santa Catarina, Southern Brazil. Phytotaxa 246(1): 49–60. https://doi.org/10.11646/phytotaxa.246.1.4

[B16] Hassemer G (2017) Taxonomic and nomenclatural notes on Neotropical *Commelina* (Commelinaceae), and an identification key for Brazil, Guyana, Paraguay, Suriname and Uruguay. Phytotaxa 303(2): 101–117. https://doi.org/10.11646/phytotaxa.303.2.1

[B17] Hertweck KL Pires JC (2014) Systematics and evolution of inflorescence structure in the *Tradescantia* alliance (Commelinaceae). Syst. Bot. 39(1): 105–116. https://doi.org/10.1600/036364414X677991

[B18] Hoehne FC (1915) Botanica. In: Historia Natural. Relatório da Commissão de Linhas Telegraphicas Estrategicas de Matto-Grosso ao Amazonas anexo 5, pt. 5: 14.

[B19] Hunt DR (1975) The Reunion of *Setcreasea* and *Separotheca* with *Tradescantia* – American Commelinaceae: I . Kew Bulletin 30(3): 443–458. https://doi.org/10.2307/4103068

[B20] Hunt DR (1980) Sections and series in *Tradescantia* – American Commelinaceae: IX. Kew Bulletin 35(2): 437–422. https://doi.org/10.2307/4114596

[B21] Hunt DR (1986a) *Campelia*, *Rhoeo* and *Zebrina* united with *Tradescantia* – American Commelinaceae: XIII. Kew Bulletin 41(2): 401–405. https://doi.org/10.2307/4102948

[B22] Hunt DR (1986b) New names and a new species in Tradescantia – American Commelinaceae: XIV. Kew Bulletin 41(2): 406 https://doi.org/10.2307/4102949

[B23] Hunt DR (2007) A new species of *Tradescantia* (Commelinaceae) from Mexico. Kew Bulletin 62(1): 141–142.

[B24] IBGE [Instituto Brasileiro de Geografia e Estatística] (2012) Manual Técnico da vegetação Brasileira: sistema fitogeográfico, inventário das formações florestais e campestres, técnicas e manejo de coleções botânicas, procedimentos para mapeamentos (2^nd^ edn), Vol. 1. IBGE, Rio de Janeiro, 272 pp.

[B25] IUCN (2001) The IUCN red list of threatened species, version 2010.4. IUCN Red List Unit, Cambridge http://www.iucnredlist.org/ [accessed: 2.6.2016]

[B26] Link JHF Otto CF (1828) Icones plantarum selectarum Horti Regii Botanici Berolinensis cum descriptionibus et colendi ratione (Vol. 2). Zehn Hefte, Berlin, 13–24. [t. 7–12]

[B27] McNeill J Barrie FR Buck WR Demoulin V Greuter W Hawksworth DL Herendeen PS Knapp S Marhold K Prado J Prud’Homme Van Reine WF Smith GF Wiersema JH Turland NJ (Eds) (2012) International Code of Botanical Nomenclature (Melbourne Code). Adopted by the Eighteenth International Botanical Congress Melbourne, Australia, July 2011. Regnum Vegetabile 154. A.R.G. Gantner Verlag KG, Sweden, 240 pp.

[B28] Panigo E Ramos J Lucero L Perreta M Vegetti A (2011) The inflorescence in Commelinaceae. Flora 206(4): 294–299. https://doi.org/10.1016/j.flora.2010.07.003

[B29] Pellegrini MOO (2015) Filogenia e revisão taxonômica de Tradescantia L. sect. Austrotradescantia D.R.Hunt (Commelinaceae). MsC thesis, Instituto de Biologia, Universidade Federal do Rio de Janeiro, Rio de Janeiro, Brazil https://doi.org/10.12705/641.3

[B30] Pellegrini MOO (2016) A new species of Tradescantia L. sect. Austrotradescantia D.R.Hunt (Commelinaceae) from Southern Brazil. Phytotaxa 265(1): 79–84.

[B31] Pellegrini MOO (2017) *Tradescantia*. Flora do Brasil 2020 em construção. Jardim Botânico do Rio de Janeiro http://floradobrasil.jbrj.gov.br/reflora/floradobrasil/FB126851 [accessed: 1.1.2017]

[B32] Pellegrini MOO Forzza RC Sakuragui CM (2015) A nomenclatural and taxonomic review of *Tradescantia* L. (Commelinaceae) species described in Vellozo’s *Flora fluminensis* with notes on Brazilian *Tradescantia*. Taxon 64(1): 151–155.

[B33] Pellegrini MOO Forzza RC Sakuragui CM (2016) (Con)Fused bracts: The identity and application of *Tradescantia cymbispatha* C.B.Clarke (Commelinaceae) and a neglected new *Tradescantia* species from Bolivia. Systematic Botany 41(4): 950–958. https://doi.org/10.1600/036364416X694053

[B34] Radford AE Dickison WC Massey JR Bell CR (1974) Vascular Plant Systematics. Harper & Row Publishers, New York, 891 pp.

[B35] Spjut RW (1994) A Systematic Treatment of Fruit Types. The New York Botanical Garden, New York, 181 pp.

[B36] Stafleu FA Cowan RS (1976) Taxonomic literature. A selective guide to botanical publications and collections with dates, commentaries and types (ed. 2, Vol. 1). Regnum Vegetabile 94. A.R.G. Gantner Verlag, Rugell, Sweden, 1136 pp.

[B37] Stafleu FA Cowan RS (1979) Taxonomic literature. A selective guide to botanical publications and collections with dates, commentaries and types (ed. 2, Vol. 2). Regnum Vegetabile 94. A.R.G. Gantner Verlag, Rugell, Sweden, 991 pp.

[B38] Stafleu FA Cowan RS (1985) Taxonomic literature. A selective guide to botanical publications and collections with dates, commentaries and types (ed. 2, Vol. 5). Regnum Vegetabile 112. A.R.G. Gantner Verlag, Rugell, Sweden, 1066 pp.

[B39] The Plant List (2013) The Plant List – Version 1.1. http://www.theplantlist.org/ [accessed: 8.12.2016]

[B40] Thiers B (2017) Index Herbariorum: A global directory of public herbaria and associated staff. New York Botanical Gardens’ Virtual Herbarium http://sweetgun.nybg.org/ih/ [accessed: 1.15.2017, continually updated]

[B41] Wade DW Evans TM Faden RB (2006) Subtribal relationships in the tribe Tradescantieae (Commelinaceae) based on rbcL and ndhF sequences. Aliso 22(1): 520–526.

[B42] Weberling F (1965) Typology of inflorescences. Botanical Journal of the Linnean Society 59: 15–221. https://doi.org/10.1111/j.1095-8339.1965.tb00058.x

[B43] Weberling F (1989) Morphology of flowers and inflorescences. Cambridge University Press, Cambridge, 1–348.

[B44] Zamudio S Espejo-Serna A López-Ferrari AR Ceja-Romero J (2013) Una nueva especie de *Tradescantia* (Commelinaceae) del estado de Querétaro, Mexico. Acta Botanica Mexicana 102: 25–30.

